# Poststroke Cell Therapy of the Aged Brain

**DOI:** 10.1155/2015/839638

**Published:** 2015-08-11

**Authors:** Aurel Popa-Wagner, Madalina Filfan, Adriana Uzoni, Pouya Pourgolafshan, Ana-Maria Buga

**Affiliations:** ^1^Department of Functional Sciences, Center of Clinical and Experimental Medicine, University of Medicine and Pharmacy of Craiova, Petru Rares Street 2, 200349 Craiova, Romania; ^2^University Hospital Rostock, Gehlsheimer Street 20, 18147 Rostock, Germany; ^3^Biochemistry Department, University of Medicine and Pharmacy “Victor Babes”, Eftimie Murgu Street 2, 300041 Timisoara, Romania

## Abstract

During aging, many neurodegenerative disorders are associated with reduced neurogenesis and a decline in the proliferation of stem/progenitor cells. The development of the stem cell (SC), the regenerative therapy field, gained tremendous expectations in the diseases that suffer from the lack of treatment options. Stem cell based therapy is a promising approach to promote neuroregeneration after brain injury and can be potentiated when combined with supportive pharmacological drug treatment, especially in the aged. However, the mechanism of action for a particular grafted cell type, the optimal delivery route, doses, or time window of administration after lesion is still under debate. Today, it is proved that these protections are most likely due to modulatory mechanisms rather than the expected cell replacement. Our group proved that important differences appear in the aged brain compared with young one, that is, the accelerated progression of ischemic area, or the delayed initiation of neurological recovery. In this light, these age-related aspects should be carefully evaluated in the clinical translation of neurorestorative therapies. This review is focused on the current perspectives and suitable sources of stem cells (SCs), mechanisms of action, and the most efficient delivery routes in neurorestoration therapies in the poststroke aged environment.

## 1. Introduction

With an increased aging population, the prevalence of age-related diseases will increase. The aging process is associated with a higher risk factor for stroke in both men and women and remains an important health issue without an accepted therapeutic strategy, except thrombolysis with recombinant tissue plasminogen activator for ischemic stroke [1, 2]. Studies before using aged animals showed that aged brains respond to stroke in a different manner when compared with young brains (e.g., with an increased blood-brain barrier permeability, a diminished antioxidant capacity or increased glial reaction, axonal sprouting, and inflammation) [3, 4]. Age-related activation of microglia in response to stroke is a key process that causes an exaggerated neuroinflammation and poor recovery after stroke [5]. However, sex differences in stroke incidence reveal that the sex ratio is reversed in very elderly people (more women than men), possibly due to their higher life expectancy, sex-related differences, and age-associated changes [6, 7]. In humans, during the aging process, many neurodegenerative disorders are associated with reduced neurogenesis and decline of proliferation of stem/progenitor cells. Stem cell based therapy is a promising modality for promoting neuroregeneration after brain injury and can be potentiated by supportive pharmacological therapy, mainly when the aging process is associated.

## 2. Neurological Recovery and Tissue Repair after Stroke Using Cell Therapy and/or Growth Factors

Stem cell therapy is focused on the functional improvement in the early stages after stroke rather than tissue replacement. Also, due to plastic capacity and tropism for damaged tissue, stem cells can be a useful tool for gene therapy in regenerative medicine [8, 9]. Cell damage after stroke involves not only neurons but also other brain cells and the extracellular matrix in a “glio-neurovascular niche” [10]. In the light of this, techniques targeting the brain cells, like growth factors or stem cell therapy, are promising tools for regenerative strategies after stroke. Some of the mechanisms involved in neuroregeneration of cell therapy after stroke are neuroprotection, axonal sprouting and regeneration, angiogenesis, and modulation of neuroinflammation. However, the mechanism of action is specific for a particular grafted cell type and the optimal delivery route, doses, or time window after lesion is still under debate.

According to their source, stem cells can be obtained from blastocyst cells (embryonic stem cells (ESCs)), adult stem cells (bone marrow derived stem cells (BMSCs) derived from peripheral blood or other tissues like adipose tissue), umbilical cord blood cells, and induced pluripotent stem cells (iPSCs). Bone marrow mononuclear cells (BM-MNCs), bone marrow derived mesenchymal stem stromal cells (BM-MSCs), umbilical cord stem cells (UCSCs), and neural stem cells (NSCs) are the most promising cells for recovery after cerebral ischemia. However, stem cells must be fully investigated for safety and therapeutic potential on animal models of neurological diseases in order to use it for clinical applications.

### 2.1. Bone Marrow Derived Cells

Bone marrow derived mononuclear cells (BM-MNCs) are a promising tool for acute stroke therapy, but most preclinical trials were performed using young animals without comorbidities. Preclinical pilot studies using autologous BM-MNCs have already been performed. In one study, Savitz and colleagues showed that the IV administration of BM-MNC extracted from the iliac crest is safe and feasible in stroke patients between 18 and 80 years old [11]. However, the aging process has a significant effect, not only on the number of BM-MSCs but also on the capacity to differentiate, survive, and repair the neuronal cell loss. Aged BM-MSCs express an aging phenotype like increased reactive oxygen species (ROS), p21 and p53 levels [12]. This phenotype can lead to less efficiency in autologous stem cell therapy using aged cells in an aging environment. In addition, the aging brain has a delay in the gene expression pattern of molecular mechanisms that promote and sustain the neuroprotection and neuroregeneration after stroke [3].

Also, the administration route has a greater impact on the biodistribution, migration, and survival of transplanted stem cells. Using single photon emission computed tomography/computed tomography (SPECT/CT) and helical CT scan after intra-arterial (IA) and intravenous (IV) administration of BM-MNCs and BM-MSCs, Mäkelä end colleagues showed that the BM-MNCs accumulated in the spleen and bones and the BM-MSCs had relatively higher uptake in the kidneys. The IA transplantation decreased the deposition of BM-MSCs in the lungs and increased uptake especially in the liver. However, both administration routes using porcine model were found to be safe [13]. Intrastriatal injections of BM-MSCs near the ischemic penumbra were found to attenuate cognitive deficits in an endothelin-1 rat model of stroke [14].

**Figure 1:**
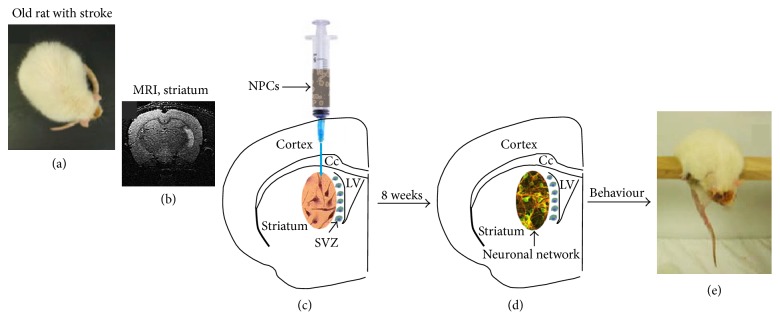
Neural stem therapy of striatal infarcts. (a) Old rat with striatal infarct. (b) Documentation of the striatal infarct by MRI. (c) Injection of NPCs into lesioned striatum. (d) Build-up of a hypothetical neuronal network weeks later. (e) Behavioural assessment of cell treatment.

Intrastriatal administration of the stem cell (SC) in the aged rats with striatal infarct leads to an elevated cell density in the ischemic area and can be monitored by magnetic resonance imaging (MRI). Injection of NPCs into lesioned striatum promotes a hypothetical neuronal network weeks later that can improve functional outcome (Figure 1).

Preclinical data suggests that autologous bone marrow derived mononuclear cell therapy attenuates the effects of inflammation in the early posttraumatic brain injury period [15].

One clinical trial indicated that the IV administration of 280.75 million BM-MNCs at 18,5 days after stroke onset is safe, but there is no beneficial effect of treatment on stroke outcome [16]. Also, Wagner and colleagues showed that the failure of autologous BM-MNCs to show a reduction of the infarct area in aged spontaneously hypertensive rats (SHR) might be because of the aging process itself and association of comorbid status [17].

### 2.2. Human Umbilical Cord Derived Cells

In the last decade, cells derived from the human umbilical cord (HUC) have emerged as a potential therapeutic alternative for stroke because of their capacity to differentiate into neural progenitor cells and their unlimited availability. Umbilical cells consist of a mixture of both hematopoietic stem cells (HSCs) and mononuclear fraction of mesenchymal stem cells (MSCs). An advantage of umbilical cord blood (UCB) derived cells is that the regulatory T-cells administration (1–5% from HUC cells) can prevent graft versus host diseases and can improve neurogenesis in the aging brain [18, 19].

Studies on HUC-derived cell population in rat model showed that the IA administration of cord blood mononuclear cells (cbMNCs) and cord blood mesenchymal stromal cells (cbMSCs) at 24 h after stroke onset is associated with a reduction in neurological deficit and infarct area [20]. Also, intravenous (IV) administration of human umbilical cord blood-derived AC133+ endothelial progenitor cells can reduce the infarct volume in rat stroke model [21]. Both IA and IV administration of UCB cells are more effective than intrastriatal administration.

cbMNCs tend to restore the reduction of brain-derived neurotrophic factor (BDNF) level after stroke, increase the glutathione peroxidase-4 (GPx-4) mRNA expression, and decrease the number of activated microglia resulting in decreased neuroinflammation and neuronal cell death and increased neurologic recovery [20, 22].

Wharton's jelly, part of the umbilical cord, contains myofibroblast-like stromal cells originated from mesenchyme that possess many unexplored advantages compared with adult stem cells. Wharton's jelly derived cells may secrete macromolecules like glycoproteins, mucopolysaccharides, glycosaminoglycans, or extracellular matrix proteins and showed capacity to differentiate into neural progenitor cells which improved neovascularization, myelination, and neurogenesis in an animal model of stroke [23–25].

However, other studies using cryopreserved cbMNC failed to demonstrate neurorestorative properties in spontaneously hypertensive rats (SHR) stroke model [26].

### 2.3. Adipose Tissue Derived Stem Cells

Adipose derived stem cells (ASCs) can be obtained from adipose tissue with minimal side effects and are able to differentiate into multiple cells that play an important role in the recovery of damaged tissue. Studies before have showed that the ASCs, in specific condition, can differentiate into the cells that express neuronal markers (NeuN and nestin) and glial markers (S100, p75) [27]. In animal model of stroke, the IV ASCs administration was founded to be safe and to improve the stroke outcome by promoting blood vessel formation and axonal sprouting by increasing the VEGF levels and decreasing apoptosis and glial scar formation [28, 29]. However, intraventricular administration was less efficient than intravascular delivery of ASCs [30].

ASCs were found to produce different growth factors like vascular endothelial growth factor (VEGF), fibroblast growth factor (FGF), platelet-derived growth factor (PDGF), and transforming growth factor (TGF-*β*) that can be increased in hypoxic conditions (e.g., stroke) [27, 29]. In addition, human adipose tissue derived MSCs are not associated with tumor formation and can be modified by genetic engineering to produce different regulatory molecules that can suppress tumor formation [31].

### 2.4. The Induced Pluripotent Stem (iPS) Cells

The recently discovered possibility to reprogram human adult somatic cells (iPS) has added a very exciting tool to the profile of the available SC and provided new perspective for neurorestorative therapy. iPS possess the potential and characters of embryonic stem cells, self-renewal and pluripotency, which allow the production of unlimited amount of precursor cells or all cell types of the body. On the other side, iPS allow the possibility to create biobanks and to avoid graft rejection. An advantage is that iPS can be obtained from blood cells and fibroblasts that are easily accessible. Chau and colleagues proved that the iPS can improve recovery after stroke in neonatal rats (P7) and adult brain by multiple mechanisms that promote angiogenesis and neurogenesis and provide essential trophic factors [32–34]. However, the mechanisms that drive cell migration, survival, and integration of grafted cell in the aged brain are still under debate. Tatarishvili and colleagues provide the first evidence that intrastriatal grafted human iPSCs decrease microglial activation and neuronal loss after stroke and improve functional deficits in aged brain after stroke [35].

Despite these exciting findings, these studies still lack proof of mechanisms of iPS that promote the improvement of functional outcome after stroke in aged brain.

### 2.5. Embryonic Stem Cell Therapy of Stroke

Although rehabilitation is important for improving functional recovery in the early stages after stroke, it does not provide a replacement for the lost neuronal cells and ultimately brain function. Neural tissue transplantation has been first explored as an experimental technique for tissue repair and functional recovery after stroke [37–39]. The techniques to achieve effective survival and growth of neuronal tissues transplanted into the CNS are now well established [40]. Most clinical studies have so far been conducted using neural tissues from human fetal donors that can better tolerate hypoxia. In contrast to mesenchymal and mononuclear stem cells, embryonic stem cells (ESCs) have an extensive capacity for self-renewal and NPCs derived from ESCs can primarily differentiate into neurons, astrocytes, and oligodendrocytes [41]. Neurosphere derived cells exert a neuroprotective action by changing the ischemic microenvironment [41]. In experimental models of stroke, however, the neuroprotective activity of NPCs derived from ESCs was mediated primarily by antireactive glial activation and proangiogenesis and to a lesser extent by differentiation into brain-specific cell types [42].

### 2.6. Combination Therapies Potentiate the Recovery Effect of Cell Therapy after Stroke

Many studies report that stem cell therapy in combination with other compounds appears to be more effective than monotherapies alone. Our group showed that the combination of BM-MSCs with granulocyte colony-stimulating factor (G-CSF) promotes angiogenesis in the ischemic area of the aged brain, without significant improvement of cognitive function [43, 44].

Association of UCB cells with G-CSF was showed to reduce neuroinflammation, enhance neurogenesis, and improve functional outcome after traumatic brain injury (TBI) [45, 46].

The study by Cui et al. revealed that a combination of subtherapeutic doses of UCB cells and simvastatin amplified endogenous angiogenesis and increased and enhanced vascular remodeling by increasing Ang1/Tie2 signalling pathway in the ischemic brain [47].

### 2.7. Stem Cells and Gene Therapy after Stroke

Gene therapy has been described as a powerful tool in treating nerve injury. Pereira and colleagues showed that the vascular endothelial growth factor (VEGF) gene and granulocyte colony-stimulating factor (G-CSF), delivered using endothelial progenitor cells (EPCs), promote regeneration and improve functional outcome [48, 49].

VEGF delivery using endothelial progenitor cells (EPCs) was found to increase migration and proliferation of human endothelial cells after ultrasonic microbubble transfection [9]. EPCs can be an efficient carrier for VEGF delivery in the ischemic area after stroke. Also, hyaluronic acid- (HyA-) based hydrogels were described to protect Sca-1(+)/CD45(−) cardiac progenitor cells (CPCs) and promote cell survival and engraftment with host tissues after transplantation [50]. Administration of gene modified SC that overexpresses neurotrophic factors like brain-derived neurotrophic factor (BDNF), Akt, and NGF can be a useful tool for neurorecovery after stroke [51–53].

## 3. Conclusion

The efficacy of stem cell therapies so far is discouragingly low mainly because the time course of interactions between host neuroinflammatory response, the main obstacle to exogenous-mediated neuronal precursor cells, and exogenously administered stem cells is still unknown. Although MSC transplantation into the brain has ascribed beneficial effects in preclinical studies of neurodegenerative or neuroinflammatory disorders [44, 54], only some studies reported that stem cells can survive in a strong neuroinflammatory environment such as an ischemic area in stroke.

**Figure 2:**
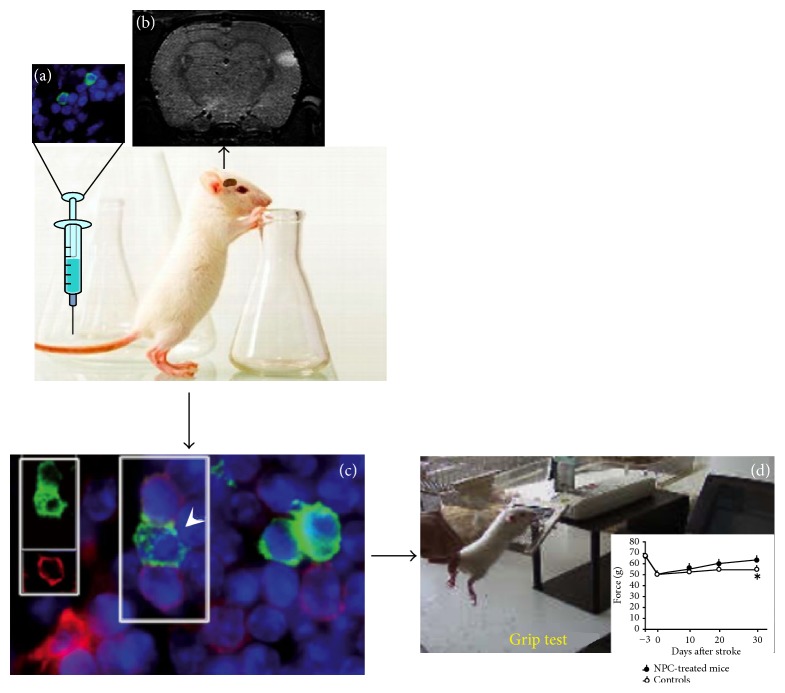
Neural stem therapy of cortical infarcts. (a) Administration of NPCs isolated from the SVZ is delivered at 72 h after reperfusion via the tail vein. (b) Documentation of the cortical infarct by MRI. (c) Visualization of transplanted cells in the peri-infarct. NPCs (arrowheads) at the ischaemic boundary zone were often detected in close proximity to phagocytes (red). Nuclei are labelled with DAPI (blue) (modified after [36]). (d) Behavioural assessment of cell treatment by the grip test.

*To conclude*, these findings strongly suggest that UCB derived cells have significant neurogenic potential but this potential has to be used in a more efficient manner to treat neurological diseases like stroke in aged people. Antineuroinflammatory therapies are a potential target to promote regeneration and repair in diverse injury and neurodegenerative conditions by stem cell therapy. Therefore, the challenge now is to determine in detail the cross talk between different populations of immune cells and grafted neural stem progenitor cells (NSPCs) at different phases after stroke in aged brain. In Figure 2, we showed that the grafted cells reach the peri-infarcted area and promote neurorecovery in the aged animals after stroke. ASCs are nontumorigenic and less immunogenic and easy to obtain with minimal side effects and are able to differentiate and express neuronal markers. However, this potential has to be further elucidated. Potential novel therapies are focused on growth factors combined with stem cells delivered together with biomaterials that can protect these active components in a hostile and aging environment. Only a few studies using exogenous grafted stem cell were performed in aged organisms. However, there remains a valuable need to understand how the complex interactions of stem cell grafts with the ischemic brain may be affected by the route, timing of cell delivery, aging environment, and, not at the end, the associated aging comorbidities.
